# Supplementary material for: The Drosophila simulans Genome Lacks the crystal-Stellate System
Source: Cells. 2022 Nov 22;11(23):3725. doi: 10.3390/cells11233725 (PMC9741089; doi:10.3390/cells11233725)
Supplement: Supplementary file 1 [file cells-11-03725-s001.zip › cells-1985778-supplementary.pdf]

D. melanogaster

Su(Ste)-specific 663 (663bp)
TCTAGATAGGGATGACGTGATTTTTTAACTCCACACAAAAGAAAGCAGAA
ATGTTTGTCCGATATATTGTTGTTCGCAATTAAACAGATTCCGCAGTGCG
TTGATATGGCCAGCTACCACCTACTAATACACCTCATACACCTCTCCTCT
AAATACTTGGTTCAAAGTGTTAGGGTCCAAGCTGGTCATATCAAGCACTC
ATTCGAGTACCATAGCATACTTTTCGTTAATAGTGGTCATAACCTGGGTA
TAATAATAAAATTAATCAAATAAAACATTCAATTCAGTTTTTTATTTAAT
TTTATGTTAAAAGAGTTCCAAAAAGTGCTTTCCTGACTACATCAAATATT
TTTTGATAATTTAATTTCTTTAAATATACAAATTCTGTTTTAATGTATTA
TAGGAGTGAGTAGAAGGTTAAAACATTATTTTCAATTTAAAAAAAAAATTA
AAATTTTGATTCCATCATTTTTATGTTATTCTTTTTGTAGAGTATTCAAT
ACTGGCAACTTCGTATTTTAGTTTGATTTCCCATGCTGTTTTTGGTTAAT
TTCCAACGTACCAGATTGATTTCGATACGTAAATTTAAACGCACTTTATT
GCCTTTTTGGAAGAACGTTGCTTCAAAACGGAAAATCGAAGGAAACCCTG
ACCGGAGAATTC

cryme3 (386bp)
TTTCCAAAAGTGTTTTCCTGACTACATCAAATATTTTTTGATAATTTAAT
TTCTTTAAATATACAAATTCTGTTTTAATGTATTATAGGAGTGAGTAGAA
GGTTAAAACACTATTTTCAATTTAAAAAAAAAATTAAAATTTTGATTCCAT
CATTTTTATGTAATTCTTTTTGTAGAGTATTCAATACTGGCAACTTCGTA
TTTTAGTTTGATTTTCCATGCTGTTTTTGGGTAATTTCCAACATACCAGA
TTGATTTTGCGCGTAAATTTAAACGCACTTTATTGCCTTTTTGGAAGAAC
GTTGCTTCAAAACGGAAAATCGAAGGAAACCCTGACCGGACCTAAGGGAC
AGCCTTGGGGGGAAAACTGGCAGTGCATTTGAATTC

D. simulans

F6.3 (471bp)
TCTAGAAGCTGCGACTCGATCCACGGCCGATACATTATGTCGAACGAGGC
GTGGATAATATGCGCAAAAGTATGAGAGACGCGACTTCGAAGTGTGTCCA
AAGCTCTCCTGTAGGCAGAAAGCCCTGCCTGTGGGCCCCAAGCGACGTGT
GTGGCAAGTCCAAAGTGAAGATCTTCTGCCCTCGCTGTAAGGACTTTTAC
GAGCTGCGATCCGATACACAGCTGGACGGAGCAATGTTCGGGACCAGCTT
CTCGCACAACTTCTTCGCGCAACTTCTTCGCGCAGCGGCCGACTGAGACC
CCAGCCGCCCCTGGAGGTCCACCGGTAAGTAATTCTATGAATTTAGTCCT
GGTTCTTTTCTACACAAAGCGCTTGCATTTGCAGTCTATTTGGCTTCCTG
TTTTTACGAGACGGCCTTGATGAAGCTCGAATCGCCCGAGTCCTCGCCCA
AGAATATCGGATCGAGAATTC

crysi3 (380bp)
TTTCCAAAAGTGTTTTCCTGACTTTACATTAAATATGTAATGATAATTTA
ATTCCAAATTTAACAGTATTCTTTAAATATATAAAACTGTATTATTCGAG
TAGGAGGTTAAAATATTACTTTCAATTTTTAATAAAATTATAATTTTGAT
TCAATCATTTTTATGTAATTCATTTTGTAGAGTATTCAATACTGGCAACT
TTGATATTGTTATTTTCCCACGATGTTTTTGGTTAATTTCCAACCTACCA

GATTGATTTCGCGCGTAAATTTATGCGCACTTTATTGCCTTTTTGGAAGA
ACGTTGCTTCAAAACGGAAAATCGAAGCAAAACCTGACCGGACCTAAGGG
ACAGCCTGGGGGAAAACTGGCAGTGCATTT

F4C2(1041bp)
GAATTCATAATAACATGAAAGATCTAATATTTTGTCATTACTAGACTAAG
AAAAATTGGAAAAAATATTAATAAATAAATTGTAATTTATTTAATATAAC
TGAGTTATAAATTTATTAATGAATTTAATGTTAAACATTTTCCTTTGGAT
ACTATTTATAAAATAATATAAAAACGCTGTGCTTGCCGCTTTGACGAGTT
TGAGATTTTTGTAAAGGCCATTCAGTACTCGACCGATTAAGGGTTACGGC
CCAACTGATACATAGATGTCATTATGGCATAGTAGGCCTTGTCATTTCGC
ACTGTACTTTTTATTATTTTTCATACGAGTAAACTGGCAACATGTCGAGC
CCGTGAGTAACTAGATTTTTCCTATGGAAAAAATAGCAAATCACAGTAAA
ATCTTGTAGCCAGAACAACAGCAGCTGGATCAGTTGGTTCCTCGGGATCA
AGGGCAACGAGTACCTCTGCCGCATGCCCATCGACTTCATTCAGGAGACG
TTCAAGATGGGCCTGGAGTACTTCACCGAGACACTGCAAGTGATACTGAA
TCCGGGGTTCGACAGTTCCTTGGACTGTGTCTTCGGCGATGAGGAGATAC
GGCATGATCCACGCCCGATACATTATGTCGGAGCGAGGCGTGGATAATAT
GCGCCAAAAGTATGAGAGACGCGACTTCGAAGTGTGTCCAAAGCTCTACT
GTAGGCAGAAAGCCCTGCCTGTGGGCCCCAGCGACGTGTGTGGCAAGTCC
AAGTGAANATCTTCTGCCCTCGCTGTAAGACTTTTACAAGCTGCGATCCG
ATCACAGCTGGACGGAGCATATGTTCGGGACCAGCTTCTCGCACAACTTC
TTAGCGCAGTGGCCGAACTTGAGACCCCAAGCCGCCACTGGACGTCCCCC
GGTAAGTAATTCGCCGAATTTAGTCCTTGTTCTTTTCTACACAAAGCGCT
TGCATTTGCAGTCAATTTGGCTTCCGGTTTTTATGAGACGGCCTTGATGA
AGCTCGAATCGCCCGAGTCCTCGCCCAAGAATATCGGATCC

20F1 (775 bp)
CCATGGGTTCCTTTAGCTCTTCCCGAAGCATTAAACAATTTAACAAAATA
TCTGGAGGCACGAAGTGTCGGACACAAGCACTCAACAATCACTTGCCTTA
TTAATCTTCTTCACACGCCGCACGCTGAATACTCTAATGACTATTATTCT
ACTATAAAGCTCATTTTTAAAATTTATTTTGTGATATTATGATTCCGGCT
TTTTCTATTTCTAAGCTATATGTAATAATATCAATGTAAAGGCAATGCAA
AACAAGAATTTTTCACATGGTGCCAATCGATCAATAATAACATAGATTTT
AAAGTCTAACACCTTCTAAGGTAAAGGGCATATTTTGTCAAATTTACAAT
GCATGAGCATACGTGTGCACACATACAGTTGTTTGCTATCACTGTATGCG
TAGAAAAGCGCTGCATTGCTGCAGCGCTCTCCGACCCTTTCGGCGCTTG*
CAAAAAATCGTATGGCGTAACGCATCTTGTTATTCTAGTGTCTATGGCCA
AACCAATGTATGTCCGTTACGCATCTTGTTATTCTAGTGTCTTTGGATGG
ACGGAGCAATGTTCGGGACCAGCTTCTCGCACAACTTCTTCGCGCAGCGG
CCGAACCCCAGCCGCCCCTGGACGTCCCCCGGTAAGTAGATTCTCCGAAT
TTAGTCCTTGTTCTTTTCTACACAAAGGCGCTTGCATTTGCAGTCTATTT
GGCTTCCGGTTTTTACGAGACGGCCTTGATGAAGCTCGAATCGCCCGAGT
CCGTCGCCCAAGAACTCTCGGATCC

17F7 (920 bp)
GGTACCCACGTCAGTAACTAGATTTTTCGTATGGAAAAAATAGCAAATCA
CAGTAAAATCTTGTAGCCAGAACAACAGCAGCTGGATCAGTTGGTTCCTC
GGGATCAAGGGCAACGAGTACCTCTGCCGCGTGCCCATCGACTACATTCA

GGAGACGTTCAACCAGATGGGCCTGGAGTACTTCACCGAGACACTGCAAG
TGATACTGAATCTGGGGTTCGACAGTTCCTTTGACTGTGTCTTCGGCGAT
GAGGAGAAGTGGTACGGCTTGATCCCCGCCCGATACATCATGTCGGAGCG
AGGCATGCCCCAAAAGTATGAGAGAGGGGACTTCGAAGTGTGTCCAAAGC
TCTCCTGTAGGCAGAAAGCCCTGCCTGTGGGCCCCAGCGACGTGTGGGTC
AAGTCCAACGTGAAGATCTTCTGCCCTCGCTGTAACGATACACAGCCGCA
CCAAAGACACTAGAATAATAAGATGAGTAACGCCATACGATTTTTTGGCA
CGCGATTTTTTGGACGTGGGCTCTATAGGTGGCTCCAAGCTCTCTCGAGT
TTTTGTTCGAGAGAGAAAGAGCGGAGAGCGCTACAGCAAACAGCTCTTTT
CTTCGCATACAGTGACAGCAGACGACTGTATGTGTTCACACGTATGCTCA
TGCATTGTAAATTTTACAAAATATGCCCTTCACCTTAGAACTTTAAATCT
ATATTATTTTTTATCAATTGGCACCATTTGAAAAATTCTTGTCTTGCATT
GCCTTAACGTTATTATTATTTTAATAAATCTTACAAATTGTAATAGACGA
ATCTATGGCACTATCCAGCCACTTTTTTGGTGAAGGCCAAATTTACTCTC
TTTCCGCTCGCTTACGCTGGGAGCGTAAGAAATCTAAAAATAGAATTTGC
TTGCTTGTGTGAGTGGATCC

F39A (3559 bp)
CTCGAGTTGCAGTCTCCATCCGAATCGCAGTACCAAGTCCAGGAGGCTTA
TCTTTTTAGACGGCTGCCGACGTGCCGGCGAAATCTTTGGGGCTGGGAAA
ATGCACTGCCAGTTTTCCCCCAGGCTGTCCCTTAGGTCCGGTCAGGTTTT
GCTTCGATTTTCCGTTTTGAAGCAACGTTCTTCCAAAAAGGCAATAAAGT
GCGCATAAATTTACGCGCGAAATCAATCTGGTAGGTTGGAAATTAACCAA
AAACATCGTGGGAAAATAACAATATCAAAGTTGCCAGTATTGAATACTCT
ACAAAATGAATTACATAAAAATTATTGAATCAAAATTATAATTTTATTAA
AAATTGAAAGTAATATTTTAACCTTCTACTCGTATAATACAGTTTTATAT
ATTTAAAGAATACTGTTAAATTTTGAATTAAATTATCATTACATATTTAA
TGTAAAGTCAGGAAAACTCTTTTGGAAGGAGGCCTTTATTAAAAAGTCTT
TTTTTTTAACATAAATTGGCTATTCCCAAAAAAAATTCCAAAGAACTATA
ATATAATAATAAGTTTTTGCATATGCATAATAAGGTAAAAATATATAATT
TTTCCCTTTTGGCCACAATTCAATTGGGAATTTATTTACGAGATTAACGA
GGTACGGCATTCCATAATTGGACGTTCATTTCAAACGTTCTGGTCAAAAC
ACTAATTATTTTTTTCAATTCAATTTTAACTATTGGTTTTTTCTGAATAA
CTTAGCTAAAAACGGTCAGGCAGTTGAAAAAAATTCGGTTTTTACTTTTA
ATAATATATTTTACGTTTTAACGAACTAAGGTGATATAATTTGTTTTTCT
AATTTTCCGAATTGGCAAAAAATGGCGAAAAACCGAACAGTTTGAACCAT
GTATTAAGGGCAGGGTAAGGGTATAAGGTGTACGAGTGTATGAGGTGTAT
TAAGTGTATGAGATGTAAGAGGTGTATAGGTCTATGAGGTGTATAAGGTG
TATGAGGTGTATGAAGTGTATGAGATGTATGAGGTGTATTAAGTGTATGA
GGGGTATAAGTAGGTGGTAGCTGGCCATATCAACGCACTGGGGAATGTGT
TTAATTGCAACAACAAGATATCGTGTGGAGTTAAACATCCAGGTTAATCC
GGGAAGGAGGAGGCGGACGAGGATCCGATACTCTCGGGCGAGGACTCGCG
CGAGGAATCGCGCTAATCGAGCGGCTTAAAGGCCGACTCATGCAACCGGA
ATCCATGGAGACTGCAAGTGCAAGCGCTTTGTGAAGAAAATAACTATACT
ATACTCGGAGAATTACTTACCGGGGGACGTACTTCTCCACGGGCGGCTGG
GGTCTCAACATCGGCAGCTCCATGAAGAACAGGTGCGGGAAGCTGGTCCC
GAACATGGCTCCGTCCAGCTTTGGACGGGATCGCGGCTTGAAGACGTCTC
TACAGCTTGGACAGTAGATCTTAACTGTGGAATGGCCCCACTCGTCGCTG
AGGCCCACTGGCAGGGCTTTCTGCCCCTTACAGTAGAACTTCGGACACAC
TCCGAAGTCCCCCCTCTCATACTTCAACGCATATCTTCCACGCCACGCTC

CGTCATAATGTAGTTGGCGTGGATCATGCCGTACAGCTTCTCCTCGTGGT
CGGAGACCGAGGCCACGGACCTGTCGACAGCGGTTCCAGGACCACGTTCA
GTGTGTCTTCCAAGTACTCCAGCCCCGCCAGTTAGCTGTTTCTTTATAGA
CTTGATGGCCCCGCGGCAGGACGAACTGTTGCCATTCATCCCACGGACGA
CATCCAGGTGGCATTTACTTTAAGGCTCGTGCTCTGGCTACAAGATTTTA
CTGTGATTGCTATATTTCCACAAGAAAAACCTAGTTACTTACGTGCTCGA
CATGTTCACTCGTTAAAAATGTAATACAAAGTGCAGTGCGAAATGACAAG
GGCTATGCCATATGCCATAATGACATCTATGTGTCAAAGTTTCGACAGGG
CTGTAACACTTAATCGGCCGGGAACTCGATAACTTTACAATAACCTCAAA
CTCGTCAAAGCGGCAAGTTCAGCTTTTATATTATTTTATTAATAGTATCC
AAAGCAATATGTTATAAATCCAGGAATTTTTTAACATTAATAAACTCAGT
TATATTACCACTTTGCTATTGAAATAAAACACAACTCTGTTTATTTTTTA
TAATAATACCCTTTACTCGTATAGTAAAAGGCTATACTAGATTCGTTGTA
AAGGCGGAAGGAATTGTTTCCGACCATATAAAGTATATATACTCTTGATC
AGGACCAATAGCCAAATCGAGCTGGCCATGTCCGTCTGTCCCTATGAACG
TCAAGATCTCAGGTAAAAGCTAAAAGGTTGAGATTCAGCATATAGATTCT
AGAGACAAAGACGCTGCGCAAGTTTGTTGACCCATGTTGCCACGCCCACA
CTAAGGTCCTCTCTAACGCCCACAAACCGCACAAAATTGTCACGCCCCCA
TTTTTGAAAAATGTGTTGATCTTTTTTTATATTTTTATTGGTCTTGTAAA
TTTTTATCTGATTACCAAGAACACAGTGGCCACGCCCATCCACCAACAAG
ACGCCCGAAATGGTCGCGTCCACACTTTTAAACAATTTTAAAAAATGTTC
TCATTATATTCCCCAATATCTATCGATATCTCACAAAAAATGATGATATC
GATATCGCATCACACTAGTTTACTCCCAATTATCCATACTAACTGTAAAT
ATTACTTTTTTACGGACTCTTAGACAAATTTTTTTTAAGAACTAGATTGT
TAGTACCTTCACATTTTCATATTGCTGGTTGGTTTTCTATAGTACAAAAA
TATAAAATGAAGCCTAGCCAACTACTAATTAAACTGTATCTTTATATTTA
ACTGGCCAAGAACCTCTAGTAACCTGTACAACCTTTTGGCAGAGTACAAA
TGGCTGAAAGCCGAAGTGGCTACAAATCGCATTTTAATAGCTGGCATGGT
CAGCGTTTCTGTTCCTTTTCGCCGTACCCTATGGCTCCTTAAGTGTGAAA
TTCAACCAGCTACTTGGAACCAAGAGGCAGGGGACTCAACGCAAGCCAAA
CCTGAAAGCGATCAGGAAAAATGTGGCTGTCAGTAAGTGCAGTGGCTCAT
AATGACTCGATGCGGCCAAGCTGAAACTGATTTAGCTATGGGATAGGAAA
TGCTATTTGGGCATGGCTGTCGATATCATTAGACGCGGATATCACGTAAT
GCCCAGAAAGTTTATACAGTGTAGAATAGAATATGCCATAACTTTATAAA
GAAACATAATTATGGATTTAAGTAATTTAAAAGACCCCTAAATAATTTGT
ACTCTGCATAAATGCTAGACTAATGGTAAACATATCATAATTTTAAAATG
AAAAAAATTTAGCACAAGGAATCCATCCACATAACGTGTGTAGTTGGTAA
ACAAGACGAACGGCTGAAAATGTTCAACTGGCGTACCGGTATAATGGCGC
AACCGTGCGAGGGAAAAATCGACGCGGCGCGCATCAAAATGCGATAGCGG
GGAGAATTC

D. sechellia

crysec3 (382bp)
TTTCCAAAAGTGTTTTCCTGACTTTACATTAAATATGTAATAATAATTTA
ATTCCAAATTTAACAGTATTCTTTAAATATATAAAACTGTATTATACGAG
TAGAAGGTTAAAATATTACTTTCAATTTTTAAAAAAATTATAATTTTGAT
TCAATCATTTTTATGTAATTCATTTTGTAGAGTATTCAATACTGGCAACT
TTGATATTGTTATTTTCCCACGATGTTTTTGGTTAATTTCCAACCTACCA
GATTGATTTCGCGCGTAAATTTATGCGCACTTTATTGCCTTTTTGGAAGA

ACGTTGCTTCAAAACGGAAAATCCGAAGCAAAACCTGACCGGACCTAAGG
GACAGCCTGGGGGAAAACTGGCAGTGCATTT

D. mauritiana

crymau3 (389 bp)
TTTCCAAAAGTGTTTTCCTGACTTTACATTAAATATGTAATGATAATTTA
ATTCCAAATTTAACAGTATTCTTTAAATATACAAATATACAAAACTGTAT
TATACGAGTAGAAGGTTAAAATATTACNTTTCAATTTTTAATAAAATTAT
AATTTTGATTCAATCATTTTTATGTAATTCATTTTGTAGAGTATTCAATA
CTGGCAACTTTGATATTGTCTTTTTCCCACGATGTTTTTGGTTAATTTCC
AACCTACCAGATTGATTTCGCGCGTAAATTTATGCGCACTTTATTGCCTT
TTTGGAAGAACGTTGCTTCAAAACGGAAAATCGAAGCAAAACCTGACCGG
ACCTAAGGGACAGCCTGGGGGAAAACTGGCAGTGCATTT

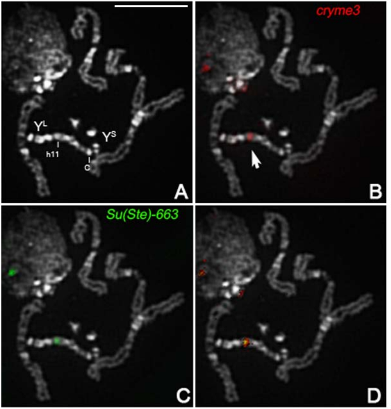
cryme3
Su(Ste)-663
Y^L
Y^S
h11
c
A
B
C
D

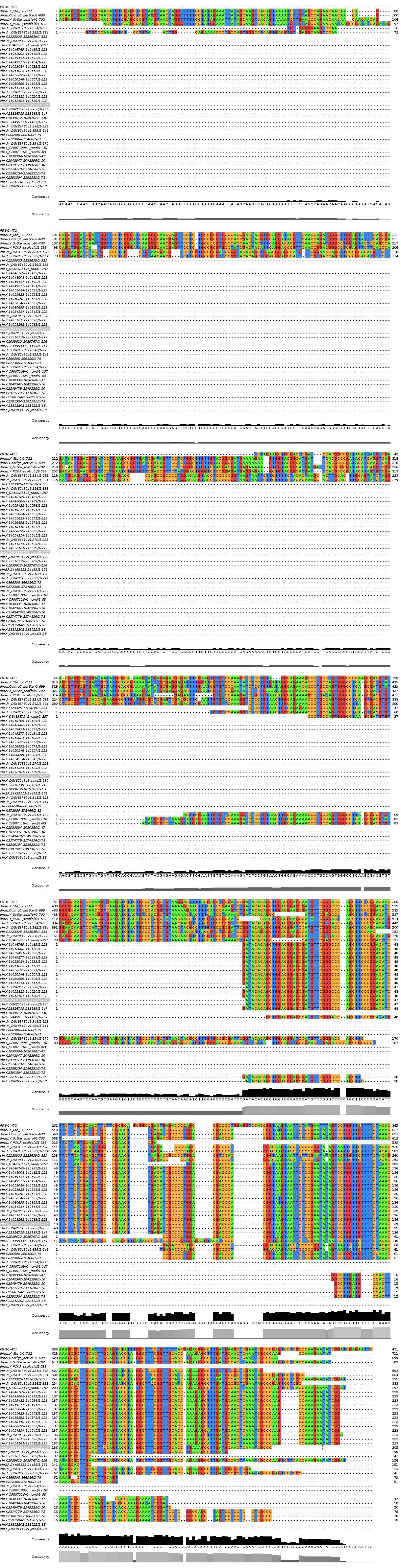
Consensus
Occupancy

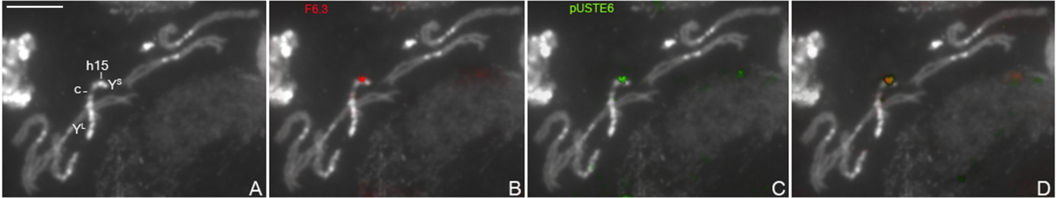

h15
Y^S
c
Y^L
A
F6.3
B
pUSTE6
C
D

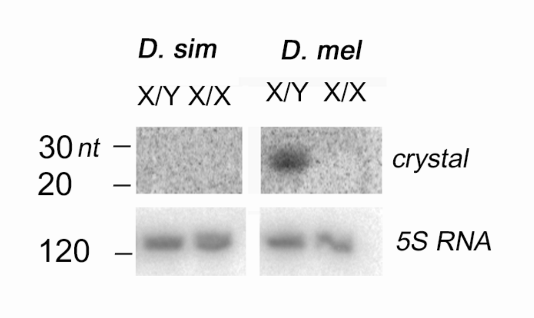

D. sim
D. mel
X/Y X/X
X/Y X/X
30 nt
20
crystal
120
5S RNA

Supplementary Materials:

Figure S1. Sequences of the clones obtained from the indicated Drosophila species.

Figure S2. FISH of the cryme3 and *Su(Ste)*-specific 663 probes on mitotic chromosomes of *D. melanogaster*. Localization by fluorescence in *situ* hybridization of cryme3-Cy2 labeled (red)(B) and *Su(Ste)*-specific 663-FluorX labeled (green) (C), on mitotic chromosomes of *D. melanogaster*, stained with DAPI. Both probes map in the h11 region of Y chromosome; (D) a merge of the two hybridization patterns; the yellow signal on the merged figure indicates the co-localization of the two probes. Centromere (c). Scale bar indicates 5 µm.

Figure S3. Multialignment of the F6.3 sequence, *Ste*, *het*^{Ste}, *Su(Ste)*, *PCKR* and the sequences of *D. melanogaster* genome obtained using the BLAT algorithm on the UCSC Genome Browser (https://genome.ucsc.edu/cgi-bin/hgBlat accessed on 28 October 2022).

Figure S4. FISH of the F6.3 and pUSTE6 probes on mitotic chromosomes of *D. simulans*. Localization by fluorescence in *situ* hybridization of F6.3-Cy2 labeled (red)(B) and pUSTE6-FluorX labeled (green) (C), on mitotic chromosomes of *D. simulans*, stained with DAPI. Both signals are in the h15 region of the Y chromosome; (D) a merge of the two hybridization patterns; the yellow signal on the merged figure indicates the co-localization of the two probes. Only the strong hybridization signal is shown. Centromere (c). Scale bar indicates 5 µm.

Figure S5. piRNA detection by Northern Blot analysis. Enriched small RNA extracts from testes and ovaries of the indicated genotypes, hybridized with the oligonucleotide named crystal. 5S RNA was used as a loading control.

Table S1. Sequence similarity between the F6.3 sequence and the orthologous sequences of *D. melanogaster*.

Sequence	Identity vs F6.3 (%)
F6.3/1-471	100
dmel.X_Ste_1/1-711	71
dmel.Contig5_hetSte./1-699	71
dmel.Y_SuSte_scaffol/1-702	68
dmel.Y_PCKR_scaffold/1-539	70
chrUn_DS483780v1:334/258-593	76
chrUn_DS483780v1:362/314-664	75
chrY:1123325-1123659/1-335	79
chrUn_DS485490v1:316/1-333	78
chrY_DS483875v1_rand/1-297	69
chrX:14046769-140469/1-223	75
chrX:14048008-140482/1-223	75
chrX:14059431-140596/1-223	75
chrX:14049277-140494/1-223	75
chrX:14053084-140533/1-223	75
chrX:14055623-140558/1-223	75
chrX:14056892-140571/1-223	75
chrX:14050546-140507/1-223	74
chrX:14060699-140609/1-223	74
chrX:14054354-140545/1-223	75
chrUn_DS485610v1:270/1-223	75
chrX:14051815-140520/1-223	74
chrX:14058161-140583/1-223	74
chrX_DS483995v1_rand/1-205	75
chrX_DS485049v1_rand/1-190	75
chrX:23316759-233169/1-147	78
chrY:1039612-1039767/1-156	80
chr2R:24499551-24499/1-151	75
chrUn_DS483780v1:348/1-123	71
chrUn_DS485490v1:699/1-141	75
chrY:866508-866586/1-79	84
chrY:872386-872466/1-81	84
chrUn_DS483780v1:394/1-170	82
chrY_CP007109v1_rand/1-187	62
chrY_CP007116v1_rand/1-83	74
chrY:1030334-1030380/1-47	76
chrY:1042347-1042396/1-50	84
chrY:2549479-2549528/1-50	82
chrY:2574779-2574856/1-78	71
chrY:2586154-2586231/1-78	71
chrY:2591504-2591581/1-78	71
chrX:23352202-233522/1-38	94
